# Temporal effects of barbiturate coma on intracranial pressure and compensatory reserve in children with traumatic brain injury

**DOI:** 10.1007/s00701-020-04677-z

**Published:** 2020-12-19

**Authors:** Fartein Velle, Anders Lewén, Timothy Howells, Pelle Nilsson, Per Enblad

**Affiliations:** Department of Neuroscience, Section of Neurosurgery, Uppsala University, SE 751 85 Uppsala, Sweden

**Keywords:** Traumatic brain injury, Children, Barbiturate coma, Refractory intracranial hypertension, Intracranial compensatory reserve

## Abstract

**Background:**

The aim was to study the effects of barbiturate coma treatment (BCT) on intracranial pressure (ICP) and intracranial compensatory reserve (RAP index) in children (< 17 years of age) with traumatic brain injury (TBI) and refractory intracranial hypertension (RICH).

**Methods:**

High-resolution monitoring data were used to study the effects of BCT on ICP, mean arterial pressure (MAP), cerebral perfusion pressure (CPP), and RAP index. Four half hour long periods were studied: before bolus injection and at 5, 10, and 24 hours thereafter, respectively, and a fifth tapering period with S-thiopental between < 100 and < 30 μmol/L. S-thiopental concentrations and administered doses were registered.

**Results:**

Seventeen children treated with BCT 2007–2017 with high-resolution data were included; median age 15 (range 6–17) and median Glasgow coma score 7 (range 3–8). Median time from trauma to start of BCT was 44.5 h (range 2.5–197.5) and from start to stop 99.0 h (range 21.0–329.0). Median ICP was 22 (IQR 20–25) in the half hour period before onset of BCT and 16 (IQR 11–20) in the half hour period 5 h later (*p* = 0.011). The corresponding figures for CPP were 65 (IQR 62–71) and 63 (57–71) (*p* > 0.05). The RAP index was in the half hour period before onset of BCT 0.6 (IQR 0.1–0.7), in the half hour period 5 h later 0.3 (IQR 0.1–0.7) (*p* = 0.331), and in the whole BCT period 0.3 (IQR 0.2–0.4) (*p* = 0.004). Eighty-two percent (14/17) had favorable outcome (good recovery = 8 patients and moderate disability = 6 patients).

**Conclusion:**

BCT significantly reduced ICP and RAP index with preserved CPP. BCT should be considered in case of RICH.

## Introduction

Despite modern neurointensive care (NIC), traumatic brain injury (TBI) remains a worldwide cause of mortality and morbidity, and treatment strategies remain challenging [35]. TBI is also common in children who require particular concern in many respects [7]. Barbiturate coma treatment (BCT) of refractory intracranial hypertension (RICH) is an established treatment option in adult TBI patients [8, 20, 23, 24, 29]. In children BCT is not as well established as in adults [19, 27, 33]. In a recent study we found that high-dose BCT in children with RICH was effective without causing unacceptable concomitant side effects and that the long-term clinical outcome was relatively favorable [37]. It is important to gain more knowledge about the intracranial pressure dynamics in pediatric TBI when BCT is used. Children have several anatomical and physiological differences compared with adults [11, 13], e.g., regarding optimal cerebral perfusion pressure (CPP) levels, cerebrovascular pressure reactivity, and intracranial compliance [2, 3, 12, 18, 28]. Intracranial compliance is the ability to compensate for increases in intracranial volume, i.e., cerebrospinal compensatory reserve. This can be expressed by the RAP index, which is based on the correlation between the amplitude of the ICP pulse wave and mean ICP [5]. There have been several studies of intracranial compliance using RAP index in adult patients with TBI [1, 5, 16, 36, 39], but to our knowledge, there has not been any study yet published regarding intracranial compensatory reserve using RAP index in children with TBI. In the third edition of Brain Trauma Foundation Guidelines for the management of Traumatic Brain Injury [19], the gathered knowledge about BCT has not increased.

The aim of this investigation was to study the effects of BCT on ICP, mean arterial pressure (MAP), CPP, and intracranial compensatory reserve (RAP index) in TBI children with RICH by analyzing these parameters at time intervals before, during, and after onset of BCT. To date no published studies have shown the effects BCT on intracranial compliance in children with TBI during consecutive intervals from before given boluses until steady state [19].

## Materials and methods

### Patients and clinical data

The Department of Neurosurgery, Uppsala University Hospital, serves the middle part of Sweden with a catchment area of about 2 million people. All neurotrauma cases in both children and adults that require neurointensive care (NIC) are admitted from the local hospitals in the region. During 2007–2017, 72 children ≤ 17 years with TBI and Glasgow Coma Scale motor score (GCSm) ≤ 5 were treated at our NIC unit. Twenty-four (33%) of these patients received BCT due to RICH. In 17 of these children high-resolution ICP data were recorded while in the remaining seven children only minute-by-minute data were collected or data were incomplete. In this retrospective study, the 17 children with high-resolution data were included.

The following clinical variables were studied: cause of injury; time from trauma to initiation of BCT; Glasgow coma scale (GCS) sum score and GCS motor score (GCSm) at admission to the NIC-unit, and GCSm at departure; the Rotterdam CT score of initial brain CT scan [21, 22]; length of BCT, given doses of thiopental and serum thiopental concentrations. Outcome was registered at 6 months, according to Glasgow outcome scale (GOS).

### Neurointensive care protocol

All patients were treated according to a standardized escalated ICP/CPP-based management protocol with specific focus on identifying and treating secondary insults, described in detail elsewhere [9, 26]. The management protocol is slightly modified in children. Briefly, the escalated management steps are as follows.

#### Basal management

All patients not responding to commands (GCSm ≤ 5) are routinely intubated and received an ICP device, either intraventricular catheter (Smiths Medical®) or intraparenchymal probe (Codman®). Mild hyperventilation (pCO_2_ 30–34 mmHg/4–4.5 kPa) is applied initially, which is gradually changed to normoventilation as soon as ICP permits. Neurologic state (GCSm) is assessed regularly by wake-up tests if the patient has stable ICP. Sedation is maintained with infusions of propofol in adults (Propofol-LipuroB; Braun Medical, Danderyd, Sweden) and midazolam in children (< 15 years of age), with morphine (Morfin Media; Media, Sollentuna, Sweden) as analgesic. The primary goals are normovolemia with adequate colloid osmotic pressure (infusions of Albumin 20% when needed and zero or slight negative water balance), normal electrolytes, and central venous pressure at 0–5 mmHg. Hypotension is treated sequentially with volume substitution and dobutamine or noradrenaline. The goal is to keep ICP < 20 mmHg, CPP around 60 mmHg in adults and as low as 45–50 mmHg in children depending on age. In the case of high ICP, increasing blood pressure above normal levels to achieve an adequate CPP alone is not considered sufficient. Spontaneous CPP levels over 60 mmHg are not actively lowered unless the raised CPP has a detrimental effect on ICP levels. The aim of this strategy is to prevent development of secondary brain edema in tissue with disturbed blood brain barrier and/or cerebral vascular autoregulation[10]. In the case of increased ICP, intermittent drainage of small volumes (1–2 ml) of cerebrospinal fluid (CSF) from an EVD is used if there is no radiologic sign of mass lesions. The intermittent drainage of CSF is after some time (1–2 days) changed to continuous drainage against a pressure level of 15–20 mmHg when the risk of developing mass lesions is lower. If ICP increases despite the basal management, a re-evaluation of occurrence of secondary insults is done, and a new CT scan is performed to rule out the presence of a mass lesion requiring surgical treatment before escalation of management protocol to the next level.

Step 1. No wake-up tests are performed. To relieve stress, more sedatives and analgesics are given, and β1-antagonist (Seloken; AstraZeneca, Södertelje, Sweden) and α2-agonist (Catapresan; Boehringer Ingelheim, Stockholm, Sweden) are administered. If these measures do not alleviate ICP problems, management is escalated further.
Step 2. BCT is initiated if cerebral CT shows no significant mass lesion with midline shift. Osmotherapy is not included in the protocol. Thiopental (Pentocur®, Abcur AB, Hälsingborg, Sweden) is used as a mono-sedative. The lowest dose needed to decrease ICP is administrated without the specific intention of obtaining burst suppression on EEG. The EEG monitoring is primarily used to observe if more thiopental can be given to decrease ICP when needed without causing unacceptably long suppression periods. The aim is to decrease ICP while minimizing the risk of medication related complications [4, 9].

BCT is initiated with a thiopental bolus (total 4–8 mg/kg) given as repeated 50-mg bolus injections in adults and repeated bolus injections of 0.7 mg/kg (total 3–5 mg/kg) in children until ICP normalizes. Repeated intermittent bolus injections are initially given to rapidly decrease ICP while ensuring that there is no substantial negative effect on mean arterial blood pressure. A continuous infusion of thiopental is started at the same time as the bolus injections are given with an initial dose of 5–10 mg/kg/h which is adjusted over time to the lowest possible dose sufficient to achieve the ICP target. The dose may also be reduced if there are unacceptably long EEG burst suppression episodes, or if serum concentrations of thiopental exceed 300 μmol/L, due to the risk of complications. During BCT, CPP levels as low as 50 mmHg are accepted in adults and 40–45 mmHg in children. Patients receive only parenteral nutrition during BCT. Patients are kept on thiopental as short as possible depending on clinical status, interpretation of intracranial dynamics (mean ICP, amplitude and plateau waves), CT brain findings, and severity of complications. If adequate doses of thiopental do not reduce ICP below 20 mmHg or BCT is not tolerated by the patient due to side effects, the next management step is initiated.

Step 3. Decompressive craniectomy (DC) is performed [38]. If there is diffuse swelling without midline shift, a bifrontotemporal DC with duraplasty is done with sparing of a bone ridge in the midline. If there is a diffuse focal mass lesion with midline shift that cannot be relieved by evacuation of a localized hematoma or confluent contusions, a large hemicraniectomy with duraplasty is performed instead. The latter may also be performed at earlier stages under such conditions.

### Neurointensive care monitoring, data collection, and analysis

The ICP waveform data are recorded at a sampling rate of 100 Hz using the Odin software for multimodality monitoring [15, 32]. The RAP index (R stands for the correlation (Pearson’s R) between Amplitude and Pressure) was calculated as the correlation over a 5-min window of each ICP pulse amplitude with the mean ICP calculated over a 10-s window centered on the peak. The moving 5-min window was advanced in 12-s increments, so that five correlation values are produced per minute [1, 5, 6, 16]. A RAP index close to 0 indicates lack of synchronization between these two parameters; a change in volume produces no or very little change of pressure, which denotes a good pressure-volume compensatory reserve (usually at low ICP levels). On the other hand, when RAP rises to +1, amplitude varies directly with ICP which denotes a low compensatory reserve. With further rise in volume, ICP rises rapidly and eventually the amplitudes decrease, and RAP values become negative. This happens when the cerebral autoregulation capacity is exhausted, cerebral arterioles passively collapse instead of dilating as a response to decreased perfusion.

**Fig. 1:**
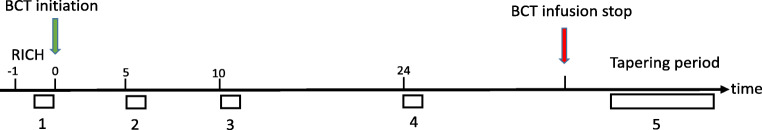
Five periods were studied: **1**. Half-hour period just before bolus of thiopental due to RICH, **2**. Between 5 and 5.5 hours after given bolus, **3**. Between 10 and 10.5 hours after given bolus, **4**. Between 24 and 24.5 hours after given bolus, and **5**. A tapering period during S-thiopental < 100 until <30. *BCT: barbiturate coma treatment. RICH: refractory intracranial hypertension.*

Mean arterial blood pressure (MAP), ICP, CPP, and RAP index were analyzed at half hour periods before, during, and after barbiturate coma treatment: (1) The period just before bolus initiation of thiopental, (2) between 5 and 5.5 hours after given bolus, (3) between 10 and 10.5 hours after given bolus, (4) between 24 and 24.5 hours after given bolus, and (5) a tapering period from when the serum thiopental concentration was below 100 until < 30 (Fig. 1) was also studied. The proportions of monitoring time (%) with ICP > 20 mm Hg and ICP > 25 mm Hg were analyzed during period 1 and the whole BCT period.

### Statistical methods

IBM, SPSS v.26, was used to perform non-parametric statistics. The Wilcoxon Signed Rank Test was used to analyze differences between the period just before bolus initiation of thiopental and each subsequent study period. Differences were considered statistically significant if *p* < 0.05.

## Results

### Patients and NIC management

Information of each individual child are presented in Table 1. The median age of the 17 children studied was 15 years (range 6–17) and median weight 59 kg (range 22-82). At admittance to Uppsala, the median GCS was 7 (range 3–8) and median GCSm was 5 (range 2–5). Median Rotterdam classification of the first CT brain was 4 (range 3–5).

**Table 1:** **Patient characteristics**: age, weight, cause of trauma, GCS sum score (GCS), and GCS motor score (GCSm) at admission and GCSm at departure, CT brain classification of first scan according to Rotterdam, Glasgow outcome scale (GOS), type of surgical intervention (decompressive craniectomy and/or evacuation of mass lesion), and time relation to barbiturate coma treatment (BCT). Median values at bottom line

Patient	Age (y)	Weight (Kg)	Cause	GCS adm	GCSm adm	CT class	GCSm dep	GOS	DC	Evacuate mass lesion
1	6	22	Sign	7	5	4	6	5	N	N
2	7	24	MVO	3	2	5	5	4	Start BCT	ASDH + DC start BCT
3	11	35	Fall	7	5	3	6	5	BCT period 3–4	N
4	11	44	Ped	7	3	4	6	4	N	N
5	13	53	Sport	8	5	4	6	5	N	ICH before BCT
6	13	47	Ped	7	5	4	6	5	N	EDH before BCT
7	14	61	Sport	5	3	4	3	3	N	EDH before BCT
8	14	59	Sport	7	5	4	6	5	N	EDH + ASDH before BCT
9	15	62	MVO	6	4	4	6	5	BCT period 4–5	N
10	15	82	Ped	7	5	4	6	4	Before BCT	Contusion + DC before BCT
11	15	55	Sport	8	5	4	D	1	N	N
12	16	65	MVO	4	2	5	2	2	Before BCT	N
13	16	80	MVO	7	5	5	5	4	N	N
14	16	60	Ped	6	4	4	6	5	BCT period 5	ICH + DC BCT period 5
15	16	58	MVO	7	4	5	6	4	N	N
16	16	64	MVO	6	3	5	4	4	BCT period 4–5	N
17	17	61	Sport	8	5	4	6	5	N	N
Median	15	59		7	5	4	6	4		

*DC* decompressive craniectomy, *EDH* epidural hematoma, *ASDH* acute subdural hematoma, *ICH* intracerebral hematoma, *MVO* motor vehicle occupant, *Ped* pedestrian, *Sign* hit by moving object

DC was performed in seven children: two acutely before BCT (patients 10 and 12), one acutely at start of BCT (patient 2), and the remaining four during BCT (patients 3, 9, 14, and 16). Three of the seven had hematoma evacuation at the same time as the DC (patient s 2, 10, and 14)

DC was performed in seven children; two acutely before BCT (patient 10 and 12), one acutely at start of BCT (patient 2), and the remaining four during BCT (patient 3, 9, 14 and 16). Three of the seven children had hematoma evacuation at the same time as the DC; one before BCT, one at start of BCT, and one in BCT period 5 (patients 2, 10, 14, respectively). In another four children, a hematoma was evacuated prior to barbiturate coma without DC (patient 5, 6, 7, 8). Ten children had Codman intraparenchymal device inserted, and three of them also received EVD (two prior to and one after BCT). The remaining seven children received EVD alone. ICP measurement continued at a median of 14 days (range 5–33).

### Barbiturate coma treatment

**Fig. 2:**
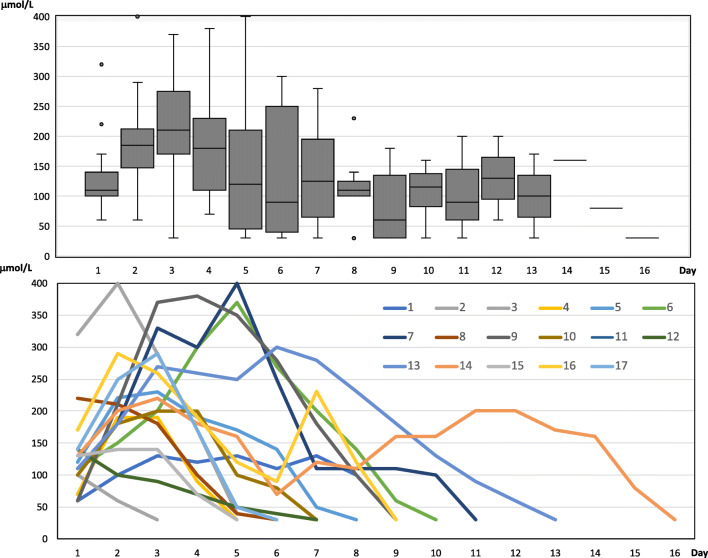
Daily serum thiopental concentrations (μmol/L); for all patients each day (upper boxplot), and for every patient (by color) each day (lower)

The BCT is summarized in Table 2. Median duration from trauma to onset of BCT was 44.5 hours (range 2.5–197.5), from start to stop 99 hours (range 21–329), and from bolus until S-thiopental < 100 117 hours (range 25–320), respectively. The tapering period with S-thiopental between < 100 and < 30 (study period 5) had a median length of 24.5 hours (range 10–95.5). Median given thiopental bolus was 4.4 mg/kg (range 2.3–14.3) followed by a mean infusion rate first 24 hours of 5.2 mg/kg/h (range 1.2–13.2). The first S-thiopental concentration was taken at median 13 h (range 3–22) after bolus, and in these samples S-thiopental was > 100 μmol/L in 13 patients (76%). In four patients this serum concentration was achieved at day two after bolus. The median max S-thiopental was 220 μmol/L (range 90–400), which was achieved by median day three of BCT (range 1–6 days). Daily serum thiopental concentrations for all and individual children are shown in Fig 2.

**Table 2:** **Barbiturate coma treatment (BCT):** Duration from trauma to thiopental bolus, duration of thiopental infusion, duration BCT from bolus to [S-thiopental] < 100, duration of tapering period, initial bolus doses of thiopental, total given dose of thiopental (including bolus) first 24 hours, max serum concentration of thiopental, day of max S-thiopental and median S-thiopental during BCT for each patient

Patient	Dur (h) TBI to thiopental bolus	Dur (h) thiopental infusion	Dur (h) BCT, until S-level < 100	Tapering period (h), interval 5	Thiopental bolus (mg/Kg)	Total dose first 24 h (mg/kg/h)	Max S-thiopental (μmol/L)	Day of max S-thiopental	Median S-thiopental and IQR
**1**	46.0	187.5	187.5	21.0	5.5	1.2	130	3	110 (100–130)
**2**	2.5	21.5	48.0	12.5	5.2	4.6	100	1	60 (45–80)
**3**	8.5	30.0	75.0	48.0	14.3	13.2	400	2	230 (73–313)
**4**	94.5	70.7	86.0	24.5	3.4	4.1	190	2	90 (70–190)
**5**	197.5	121.0	147.0	24.5	9.2	6.6	230	3	155 (103–198)
**6**	24.0	118.5	179.0	24.0	3.2	4.2	370	5	175 (118–253)
**7**	44.5	114.2	201.0	24.0	9.8	5.4	400	5	110 (110–275)
**8**	68.2	96.6	92.0	47.5	7.6	5.2	220	1	140 (55–203)
**9**	41.3	127.0	179.0	24.0	2.8	4.5	380	4	210 (100–350)
**10**	11.0	99.0	117.0	48.0	3.0	6.3	200	3	100 (90–190)
**11**	78.0	25.0	25.0	10.5	3.6	3.4	90	1	90
**12**	30.8	21.0	64.0	95.5	2.3	3.3	140	1	70 (45–95)
**13**	94.0	170.5	258.0	48.0	4.4	5.8	300	6	180 (110–260)
**14**	124.0	329.0	320.0	48.0	3.3	6.4	220	3	160 (118–185)
**15**	68.0	66.0	116.0	10.0	6.0	5.5	140	2	130 (70–140)
**16**	37.0	163.5	181.0	24.0	6.6	5.7	290	2	167 (120–230)
**17**	35.0	75.0	94.0	48.0	4.1	4.5	290	3	155 (73–230)
**Median**	44.5	99.0	117.0	24.5	4.4	5.2	220	3	130 (80–200)

### Effects of barbiturate coma treatment

**Fig. 3:**
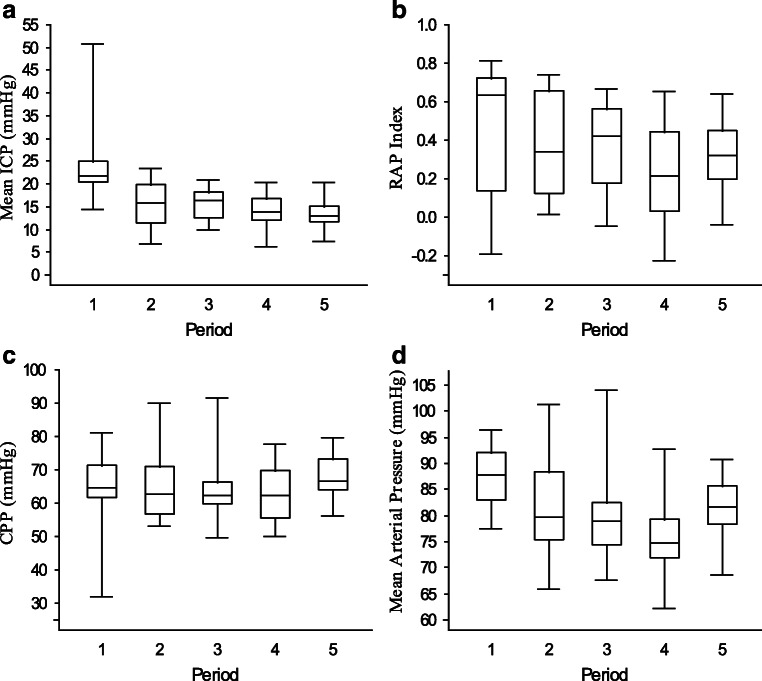
Box plots visualizing ICP **a**, RAP index **b**, CPP c, and MAP **d** during the five periods shown in Fig 1 (values in Table 3)

The temporal changes in median ICP, RAP index, CPP, and MAP, respectively, for the five consecutive time periods (Fig. 1) are shown in Table 3 and Fig. 3. Median ICP was 22 (IQR 20–25) in the half hour period before onset of BCT and 16 (IQR 11–20) in the half hour period 5 h later (*p* = 0.011). The RAP index was in the half hour period before onset of BCT 0.6 (IQR 0.1–0.7), in the half hour period 5 h later 0.3 (IQR 0.1–0.7) (*p* = 0.331), and in the whole BCT period 0.3 (IQR 0.2–0.4) (*p* = 0.004). Looking at proportions of time spent above the defined thresholds, ICP was >20 mmHg in 70% of the time and > 25 mmHg in 33% of the time in period 1 and there were significantly lower values for the whole BCT period; ICP > 20 mmHg 7% of the time (*p* = 0.002) and ICP 25 mmHg 0.5% of the time (*p* = 0.002). Median MAP decreased after initiation of BCT (Fig 3d). Median CPP remained more or less unchanged; just above 60 mm Hg before onset of BCT and during the first 24 hours of BCT, and slightly increased during the tapering period. All children received inotropic infusions (noradrenaline or dobutamine) during BCT.

**Table 3:** **Intracranial monitoring and the effects of barbiturate coma treatment:** Median values and inter quartile ranges (IQR) of ICP, RAP, MAP and CPP from five consecutive time periods just before and during BCT as described in Fig. 1. Last column presents the whole BCT period. Wilcoxon Signed Rank Test was used to analyze differences between the period just before bolus initiation (period 1) of thiopental and each subsequent study period

Median (IQR)	Period 1 *n* = 14	Period 2 *n* = 15	Period 3 *n* = 16	Period 4 *n* = 15	Period 5 *n* = 16	Whole BCT *n* = 17
ICP	22 (20–25)	16 (11–20) *p* = 0.011	16 (12–18) *p* = 0.004	14 (12–17) *p* = 0.002	13 (10–15) *p* = 0.002	14 (13–15) *p* = 0.003
RAP	0.6 (0.1–0.7)	0.3 (0.1–0.7) *p* = n.s.	0.4 (0.2–0.6) *p* = n.s.	0.2 (0.0–0.4) *p* = 0.046	0.3 (0.3–0.4) *p* = n.s	0.3 (0.2–0.4) *p* = 0.004
MAP	88 (83–92)	80 (75–88) *p* = n.s.	79 (74–82) *p* = n.s.	75 (72–79) *p* = 0.023	82 (78–85) *p* = n.s.	78 (75–80) *p* = n.s.
CPP	65 (62–71)	63 (57–71) *p* = n.s.	62 (60–66) *p* = n.s.	62 (55–69) *p* = n.s.	74 (66–81) *p* = 0.046	65 (63–67) *p* = n.s.

### Clinical outcome

When leaving the NIC unit, the median GCSm was 6 (range 1–6) compared with 5 (range 2–5) at admission (Table 1). Median GOS after 6 months was 4 (range 1–5) (Table 1 and Fig. 5). Patient 11 died due to cardiac arrest caused by sepsis 5 days after trauma and 10.5 hours after discontinuation of a 25 hours long BCT period (thiopental 3.4 mg/Kg/h and max S-thiopental 90). Patient 12 remained vegetative and died 2 years after trauma.

## Discussion

**Fig. 4:**
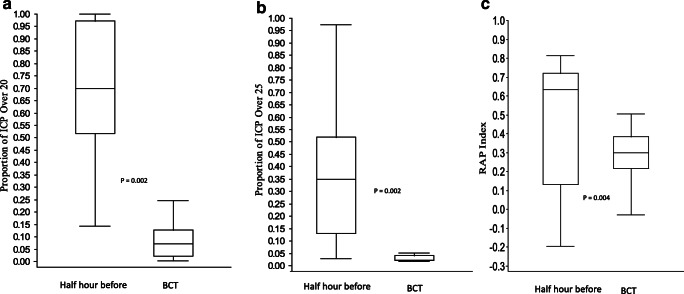
Proportion of ICP over 20 mm Hg **a**, proportion of ICP over 25 mm Hg **b,** and RAP index **c** for half hour before onset of barbiturate coma treatment (BCT) and for the whole BCT period. Wilcoxon Signed Rank Test was used to analyze differences between the periods

**Fig. 5:**
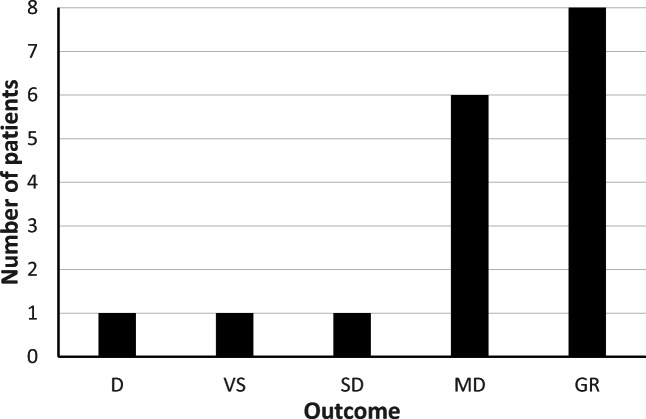
Outcome 6 months after TBI according to Glasgow outcome scale (GOS). N = 17 (D death; VS vegetative state; SD severe disability; MD moderate disability; GR good recovery).

BCT is well established in adult TBI patients, but there are only few published clinical studies of BCT in children [19]. We recently reported relatively favorable results and acceptable side effects in our experience, when BCT was applied as a late treatment in an escalated management protocol [37]. The aim of the present study was to analyze in detail the effects of BCT on ICP and intracranial compensatory reserve (RAP index) during mono-sedative BCT in 17 TBI children with RICH. The major findings were that both ICP and RAP index improved substantially shortly after initiation of BCT with a slight decrease of MAP but virtually unchanged CPP (Fig 4) and that 82% of the children achieved favorable outcome despite the development of RICH (Fig. 5).

The administrated thiopental bolus doses and total dose given first 24 h (Table 2) appeared to be according to our treatment protocol. The individual serum concentrations varied between patients and over time but were in general highest the first days after initiation of BCT and decreased gradually thereafter (Table 2 and Fig. 2), which reflects that the thiopental administration was directed by the ICP level and not with the goal of achieving burst suppression on EEG. The maximal serum concentration was rarely > 300 μmol/L, which was recommended to avoided complications. Thus, the compliance to the protocol seems to have been good.

During the last half hour before start of BCT, median ICP was 22 cm Hg, and the median proportion of monitoring time with ICP > 20 cm Hg and ICP > 25 cm Hg was ~ 70% and ~ 30%, respectively, for that period of time. This indicates that there was a clear indication for escalating the treatment and start BCT, which was further supported by a median RAP index of 0.6 the half hour before start of BCT. The severity of RICH was also underlined by the fact that several children already had been operated with evacuation of mass lesion and decompressive craniectomy before initiation of BCT.

Looking at the early effects of thiopental on intracranial pressure dynamics, median ICP and median RAP index were clearly reduced in period 2 5 h after BCT bolus (Table 3 and Fig. 3). The improvement in ICP and RAP was maintained in the later study periods. The effects were also clear when the proportions of monitoring time the half hour before BCT with ICP > 20 mm Hg (70%) and ICP > 25 mm Hg (33%) were compared with the whole BCT period (7 and 0.5%, respectively) (Fig. 4).

BCT was not persistently sufficient treatment in 4 patients (24%) who needed surgery during BCT, one between periods 3–4 (patient 3), two between periods 4–5 (patients 9 and 16), and one during period 5 (patient 14), respectively (Table 1). However, BCT was sufficient in the large majority of the cases (76%) with RICH. The surgical treatment (DC and mass lesion evacuation) in these patients may to some extent have contributed to the results seen in the later study periods but have probably not changed the overall picture ascribed to the BCT. The overall results are strengthened by that the effects of BCT presented for periods 2 and 3 were solely an effect of the BCT, i.e., not an influence of DC or mass lesion evacuation.

A slight reduction of median MAP was seen during BCT, but median CPP remained mainly unchanged due to the ICP reduction. There was of course individual variation and CPP decreased in some patients. The reason why we accept lower CPP levels during BCT in our protocol is based on the theoretical assumption that barbiturates decrease cerebral metabolism and thereby also the oxygen demand [14, 34]. Optimal CPP is difficult to define in individual cases. Intracerebral microdialysis for metabolic monitoring of the brain and monitoring of pressure autoregulation may provide valuable information for individualized management.

The early effect of BCT on ICP is usually explained by the coupling between cerebral metabolism and cerebral blood flow; the decreased cerebral metabolism caused by the BCT lowers cerebral blood flow by vasoconstriction which reduces the cerebral blood volume and thereby also ICP [25]. Barbiturates may also improve an ischemic situation, by decreasing the metabolism and the metabolic oxygen demand [34], which untreated may lead to irreversible brain injuries and subsequent brain edema. In addition, barbiturates may also prevent secondary injury mechanisms, e.g., glutamate excitotoxicity [14]. On the other hand, BCT has been associated with severe side effects which may lead to multiorgan failure [17, 30]. However, the complications are strongly related to the serum concentration. The risk may be reduced if higher serum concentrations (> 300 μmol/L) are avoided [31]. Furthermore, when there are indications of emerging severe complications, these can be eluded if BCT is stopped and decompressive craniectomy considered as an alternative. The results of the present study and our previous study of BCT in children [37] indicate that BCT can be managed with favorable results.

### Limitations of the study

The study is based on a small retrospective heterogeneous patient material and there is no control group. However, published results on the use of barbiturates in children with TBI overall is very sparse, as can be seen in the newly updated 3rd Edition Guidelines for the Management of Pediatric Severe Traumatic Brain Injury [19], and therefore this study may provide valuable information, especially since the effect of BCT on ICP and intracranial compensatory reserve has to our knowledge not been evaluated in children before in a systematic way using high-resolution monitoring data.

## Conclusion

BCT due to RICH significantly and quickly reduces ICP and improves intracranial compensatory reserve in children with TBI, with maintained level of cerebral perfusion. By using BCT many DC can be avoided, even if BCT may be insufficient and DC needed in a small proportion of the cases. On the other hand, BCT also improved ICP and RAP in some children developing RICH after early DC. The overall clinical outcome in these patients treated with BCT due to RICH also seemed to be relatively favorable, but should be interpreted with caution due to the relatively low number of patients.

## References

[1] Balestreri M, Czosnyka M, Steiner LA, Schmidt E, Smielewski P, Matta B, Pickard JD (2004). Intracranial hypertension: what additional information can be derived from ICP waveform after head injury?. Acta Neurochir.

[2] Brady KM, Shaffner DH, Lee JK, Easley RB, Smielewski P, Czosnyka M, Jallo GI, Guerguerian AM (2009). Continuous monitoring of cerebrovascular pressure reactivity after traumatic brain injury in children. Pediatrics.

[3] Burman R, Alperin N, Lee SH, Ertl-Wagner B (2018). Patient-specific cranio-spinal compliance distribution using lumped-parameter model: its relation with ICP over a wide age range. Fluids Barriers CNS.

[4] Cordato DJ, Herkes GK, Mather LE, Morgan MK (2003). Barbiturates for acute neurological and neurosurgical emergencies--do they still have a role?. J Clin Neurosci.

[5] Czosnyka M, Guazzo E, Whitehouse M, Smielewski P, Czosnyka Z, Kirkpatrick P, Piechnik S, Pickard JD (1996). Significance of intracranial pressure waveform analysis after head injury. Acta Neurochir.

[6] Czosnyka M, Steiner L, Balestreri M, Schmidt E, Smielewski P, Hutchinson PJ, Pickard JD (2005). Concept of "true ICP" in monitoring and prognostication in head trauma. Acta Neurochir Suppl.

[7] Dewan MC, Mummareddy N, Wellons JC, Bonfield CM (2016). Epidemiology of Global Pediatric Traumatic Brain Injury: Qualitative Review. World Neurosurg.

[8] Eisenberg HM, Frankowski RF, Contant CF, Marshall LF, Walker MD (1988). High-dose barbiturate control of elevated intracranial pressure in patients with severe head injury. J Neurosurg.

[9] Elf K, Nilsson P, Enblad P (2002). Outcome after traumatic brain injury improved by an organized secondary insult program and standardized neurointensive care. Crit Care Med.

[10] Elf K, Nilsson P, Ronne-Engstrom E, Howells T, Enblad P (2005). Cerebral perfusion pressure between 50 and 60 mm Hg may be beneficial in head-injured patients: a computerized secondary insult monitoring study. Neurosurgery.

[11] Figaji AA (2017). Anatomical and Physiological Differences between Children and Adults Relevant to Traumatic Brain Injury and the Implications for Clinical Assessment and Care. Front Neurol.

[12] Figaji AA, Zwane E, Fieggen AG, Argent AC, Le Roux PD, Siesjo P, Peter JC (2009). Pressure autoregulation, intracranial pressure, and brain tissue oxygenation in children with severe traumatic brain injury. J Neurosurg Pediatr.

[13] Figaji AA, Graham Fieggen A, Mankahla N, Enslin N, Rohlwink UK (2017). Targeted treatment in severe traumatic brain injury in the age of precision medicine. Childs Nerv Syst.

[14] Goodman JC, Valadka AB, Gopinath SP, Cormio M, Robertson CS (1996). Lactate and excitatory amino acids measured by microdialysis are decreased by pentobarbital coma in head-injured patients. J Neurotrauma.

[15] Howells T, Elf K, Jones PA, Ronne-Engstrom E, Piper I, Nilsson P, Andrews P, Enblad P (2005). Pressure reactivity as a guide in the treatment of cerebral perfusion pressure in patients with brain trauma. J Neurosurg.

[16] Howells T, Lewen A, Skold MK, Ronne-Engstrom E, Enblad P (2012). An evaluation of three measures of intracranial compliance in traumatic brain injury patients. Intensive Care Med.

[17] Kasoff SS, Lansen TA, Holder D, Filippo JS (1988). Aggressive physiologic monitoring of pediatric head trauma patients with elevated intracranial pressure. Pediatr Neurosci.

[18] Kiening KL, Schoening W, Unterberg AW, Stover JF, Citerio G, Enblad P, Nilssons P (2005). Assessment of the relationship between age and continuous intracranial compliance. Acta Neurochir Suppl.

[19] Kochanek PM, Tasker RC, Carney N, Totten AM, Adelson PD, Selden NR, Davis-O'Reilly C, Hart EL, Bell MJ, Bratton SL, Grant GA, Kissoon N, Reuter-Rice KE, Vavilala MS, Wainwright MS (2019). Guidelines for the Management of Pediatric Severe Traumatic Brain Injury, Third Edition: Update of the Brain Trauma Foundation Guidelines. Pediatr Crit Care Med.

[20] Lee MW, Deppe SA, Sipperly ME, Barrette RR, Thompson DR (1994). The efficacy of barbiturate coma in the management of uncontrolled intracranial hypertension following neurosurgical trauma. J Neurotrauma.

[21] Liesemer K, Riva-Cambrin J, Bennett KS, Bratton SL, Tran H, Metzger RR, Bennett TD (2014). Use of Rotterdam CT scores for mortality risk stratification in children with traumatic brain injury. Pediatr Crit Care Med.

[22] Maas AI, Hukkelhoven CW, Marshall LF, Steyerberg EW (2005). Prediction of outcome in traumatic brain injury with computed tomographic characteristics: a comparison between the computed tomographic classification and combinations of computed tomographic predictors. Neurosurgery.

[23] Marshall LF, Smith RW, Shapiro HM (1979). The outcome with aggressive treatment in severe head injuries. Part II: acute and chronic barbiturate administration in the management of head injury. J Neurosurg.

[24] Marshall GT, James RF, Landman MP, O'Neill PJ, Cotton BA, Hansen EN, Morris JA, May AK (2010). Pentobarbital coma for refractory intra-cranial hypertension after severe traumatic brain injury: mortality predictions and one-year outcomes in 55 patients. J Trauma.

[25] Nordstrom CH, Messeter K, Sundbarg G, Schalen W, Werner M, Ryding E (1988). Cerebral blood flow, vasoreactivity, and oxygen consumption during barbiturate therapy in severe traumatic brain lesions. J Neurosurg.

[26] Nyholm L, Lewen A, Frojd C, Howells T, Nilsson P, Enblad P (2012). The use of nurse checklists in a bedside computer-based information system to focus on avoiding secondary insults in neurointensive care. ISRN Neurol.

[27] Orliaguet GA, Meyer PG, Baugnon T (2008). Management of critically ill children with traumatic brain injury. Paediatr Anaesth.

[28] Philip S, Udomphorn Y, Kirkham FJ, Vavilala MS (2009). Cerebrovascular pathophysiology in pediatric traumatic brain injury. J Trauma.

[29] Rea GL, Rockswold GL (1983). Barbiturate therapy in uncontrolled intracranial hypertension. Neurosurgery.

[30] Roberts I, Sydenham E (2012). Barbiturates for acute traumatic brain injury. Cochrane Database Syst Rev.

[31] Schalen W, Messeter K, Nordstrom CH (1992). Complications and side effects during thiopentone therapy in patients with severe head injuries. Acta Anaesthesiol Scand.

[32] Signorini DF, Piper IR, Jones PA, Howells TP (1997). Importance of textual data in multimodality monitoring. Crit Care Med.

[33] Thomale UW, Graetz D, Vajkoczy P, Sarrafzadeh AS (2010). Severe traumatic brain injury in children--a single center experience regarding therapy and long-term outcome. Childs Nerv Syst.

[34] Thorat JD, Wang EC, Lee KK, Seow WT, Ng I (2008). Barbiturate therapy for patients with refractory intracranial hypertension following severe traumatic brain injury: its effects on tissue oxygenation, brain temperature and autoregulation. J Clin Neurosci.

[35] Thurman DJ (2016). The Epidemiology of Traumatic Brain Injury in Children and Youths: A Review of Research Since 1990. J Child Neurol.

[36] Timofeev I, Czosnyka M, Nortje J, Smielewski P, Kirkpatrick P, Gupta A, Hutchinson P (2008). Effect of decompressive craniectomy on intracranial pressure and cerebrospinal compensation following traumatic brain injury. J Neurosurg.

[37] Velle F, Lewen A, Howells T, Enblad P, Nilsson P (2019) Intracranial pressure-based barbiturate coma treatment in children with refractory intracranial hypertension due to traumatic brain injury. J Neurosurg Pediatr:1–9. 10.3171/2019.10.PEDS19268 10.3171/2019.10.PEDS19268 31881539

[38] Wettervik TS, Lenell S, Nyholm L, Howells T, Lewen A, Enblad P (2018). Decompressive craniectomy in traumatic brain injury: usage and clinical outcome in a single centre. Acta Neurochir.

[39] Zeiler FA, Kim DJ, Cabeleira M, Calviello L, Smielewski P, Czosnyka M (2018). Impaired cerebral compensatory reserve is associated with admission imaging characteristics of diffuse insult in traumatic brain injury. Acta Neurochir.

